# Analysis of joint mobility patterns among preschool children

**DOI:** 10.1590/S1516-31802005000300006

**Published:** 2005-05-02

**Authors:** Neuseli Marino Lamari, Alceu Gomes Chueire, José Antonio Cordeiro

**Keywords:** Joint instability, Child, Pliability, Musculoskeletal physiologic phenomena, Knee joint, Wrist joint, Metacarpophalangeal joint, Elbow joint, Instabilidade articular, Criança, Maleabilidade, Fenômenos fisiológicos musculosqueléticos, Articulação do joelho, Articulação do punho, Articulação metacarpofalângica, Articulação do cotovelo

## Abstract

**CONTEXT AND OBJECTIVE:**

Standardization of normal joint mobility criteria is complex, especially for preschool children, for whom differentiation from hypermobility (JH) is even more difficult. This study aimed to investigate joint mobility of the fifth finger, wrist, elbow, knee and trunk among preschool children, estimate the incidence of JH and evaluate the effect of gender and age and the applicability of standard criteria for identifying JH among preschool children.

**DESIGN AND SETTING:**

Cross-sectional descriptive and quantitative study, at São José do Rio Preto medical school.

**METHODS:**

1,120 healthy children (534 boys, 47.7%; 586 girls, 52.3%; age range: 4-7 years) were evaluated using Beighton scores. Passive extension of fifth finger (> 90°), passive apposition of thumb to forearm, active extension of elbow and knee (> 10°) and anterior trunk flexion placing flat hand on ground were assessed. One point was scored for each positive result (maximum: 9). Scores > 4 were considered to be JH. Student's t test and variance analysis were used for statistical analysis.

**RESULTS:**

JH was observed in 80% of wrists, 53.3% of fifth fingers, 36.6% of elbows, 14% of trunks and 12.5% of knees. Scores > 4 were found for 64.6% of the children. Females had higher angular values. Lower scores were associated with greater age.

**CONCLUSIONS:**

JH is a common condition among preschool children and currently available methods are inadequate. New parameters and criteria should be developed for identifying JH among these children.

## RESUMO

**CONTEXTO E OBJETIVO:**

A padronização dos critérios de mobilidade articular é complexa e nenhum estudo na literatura avaliou exclusivamente crianças pré-escolares, idade em que a diferenciação entre a mobilidade articular normal e a hipermobilidade torna-se ainda mais difícil. Objetivamos investigar a mobilidade articular no quinto dedo, punho, cotovelo, joelho e tronco em pré-escolares; estimar a incidência da hipermobilidade articular; avaliar o efeito da idade e do sexo nos resultados e a aplicabilidade dos critérios de Beighton e cols.^3^ para identificar a hipermobilidade articular em pré-escolares.

**TIPO DE ESTUDO E LOCAL:**

Transversal, descritivo e qualitativo, na Faculdade de Medicina de São José do Rio Preto.

**MÉTODOS:**

1.120 crianças saudáveis, 534 (47,7%) meninos e 586 (52,3%) meninas (idade entre quatro e sete anos), foram avaliadas de acordo com os escores de Beighton et al.^3^ para extensão passiva do quinto dedo (> 90°), posição passiva do polegar ao antebraço, extensão ativa do cotovelo e do joelho (> 10°) e flexão anterior do tronco (colocação das mãos espalmadas no solo). Considerou-se um ponto para cada resultado positivo. O escore máximo foi 9. Foi considerado hipermóvel a criança com escore > 4. A hipermobilidade foi estimada e comparada com a idade e o sexo. O teste t de Student e a análise da variância foram usados para análise estatística.

**RESULTADOS:**

A hipermobilidade articular foi identificada em 80% (punho), 53,3% (quinto dedo), 36,6% (cotovelo), 14% (tronco) e 12,5% (joelho). O escore > 4 foi encontrado em 64,6% das crianças, as meninas tiveram maiores valores angulares e menores escores foram associados com o aumento da idade.

**CONCLUSÕES:**

A hipermobilidade articular é uma condição comum neste grupo de idade e o método correntemente utilizado para avaliar é inadequado. Portanto, novos parâmetros e critérios devem ser desenvolvidos para identificar a hipermobilidade articular em pré-escolares.

## INTRODUCTION

Joint function and joint mobility in human beings is extremely complex and variable. It is quite difficult to describe the position of the different parts of the body during movement.

Normal joint mobility is a function of the joint capsule, ligament and muscle tone. Stretch resistance and rubbing against the tendon sheath restrain joint mobility.^1^

There is clear evidence that some factors such as gender, age group, ethnic group, certain physical activities and their intensity, and the presence of certain pathological conditions, are associated with joint mobility.^2^

Several studies have reported that females have greater joint mobility at different ages.^3-5^ However, the parameters and criteria currently used for evaluating joint mobility are the same for all age ranges, and adults and children are included in the same sample group.^3,6^ On the other hand, many studies have confirmed that mean joint scores are higher among children and adolescents than among adults.^3,7^ This suggests that the criteria and parameters used for children should be reviewed.

Physicians have investigated joint hypermobility since the time of Hippocrates, but no differentiation between normal and hypermobile joints has yet been established. There is no doubt that joint hypermobility may indicate the upper end of the "normal" range of joint movement among normal individuals.^8,9^ It is more prevalent among females^10-12^ and it is a common finding among children. Most cases are asymptomatic.^11,12^ It is usually a benign, non-pathological phenomenon, although sequelae may develop over time and lead to joint pain in healthy individuals that has no relationship with diseases of the connective tissue.^13^ This situation contrasts with cases of individuals with rheumatic diseases that lead to joint hypermobility.^14^

Most of this joint hypermobility is considered to be a result of regular physical exercise, often beginning in childhood.^15,16^ However, there are indications that this is an inherited condition that is unrelated to physical activities.^17,18^ Thus, joint hypermobility could be characterized as a genetic collagen disorder that is demonstrated by the degree of collagen cross-linking. Such linking increases the ability of the collagen to attract and hold water, and consequently increases joint mobility. Body water content decreases with age, and cross-linking between collagen molecules increases, thus decreasing joint mobility.^19^ Murray and Woo^20^ have suggested that hypermobility is a complex genetic characteristic with multiple genes for the phenotype and degree of hypermobility. On the other hand, as already highlighted in many studies (Beighton et al.,^1^ for instance), the final result suggests that there is a combination of genetic and acquired factors.

The combination of joint hypermobility and rheumatic symptoms was first reported by Sutro^21^ and was later reported by other authors as well.^22,23^Joint hypermobility syndrome is a common finding in clinical practice.

The first quantitative system for analyzing joint hypermobility was suggested by Carter and Wilkinson,^24^ and has subsequently been modified by several investigators.^3,25^ The parameters reported by Beighton et al.,^3^ which evaluate different parts of the body bilaterally, are currently the ones that are most used.^26,27^ They have also been used in the criteria proposed by the British Society for Rheumatology in 1992, to diagnose joint hypermobility syndrome.^27^ However, some investigators^28,29^ have only used upper limb parameters and, while most of them have used bilateral parameters,^3,8,30^ others have used unilateral parameters.^31,32^

There are no objective and well-defined evaluation criteria for joint hypermobility in children. This results in major and insurmountable difficulties in making comparisons between the results from the different studies already carried out. Characterization of joint mobility in normal populations, using a scoring system that objectively identifies the different levels of joint mobility is required.

This study was aimed at investigating joint mobility in groups of kindergarten children, to characterize the sample in relation to the mobility of the fifth finger, wrist, elbow, knee and trunk using several parameters. It also aimed to estimate the incidence of hypermobility for each of the studied joints, evaluate the effect of gender and age on each of the parameters, and evaluate the Beighton et al.^3^ parameters and methodological criteria, in order to establish what degree of joint hypermobility is present among preschool children.

## MATERIAL AND METHODS

The study included 1,120 healthy, asymptomatic Brazilian children of mixed racial background from March 1991 to December 1992. Their ages ranged from 4 to 7 years. There were 534 boys (47.7%) and 586 girls (52.3%). Nine preschools out of the 23 available in São José do Rio Preto, State of São Paulo, Brazil, were randomized to participate in the study. All of the children met the inclusion criteria.

Joint mobility was evaluated according to the scores proposed by Beighton et al.^3^ for five body areas. One point was scored for each positive result (for each side), and scores of 4 or more points were considered to indicate hypermobility. Passive extension of the fifth finger, apposition of thumb to forearm, active extension of the elbow and knee and active anterior flexion of the trunk with passive extension of the knees, with hands flat on the ground, were evaluated.

Passive hyperextension of the fifth finger was defined as present when the angle was greater than 90°. The measurement of the angle was obtained by placing a angle measuring device (goniometer) and the hand on a flat surface, the finger was passively extended away from the surface with the support of the middle and the distal phalanges until resistance was observed. The angle formed by finger and hand was registered.

Hypereflexion of the wrist was defined by the touch of the thumb on the forearm. The thumb was passively stretched by the examiner towards the flexing part of the forearm, but with the forearm extended.

Active hyperextension of the elbow was defined as present when the angle was greater than 10°. The angle measurement was obtained with the arm and forearm passively extended in supine position and tilting the hand backwards. The goniometer was placed beside the hand with its axis aligned in parallel to the axis of the elbow.

Active hyperextension of the knee was defined as present when the angle was greater than 10°. The angle measurement was obtained with the knee actively extended. The child was placed in orthostatic position, with feet close together side by side. The goniometer was placed beside the knee with its axis placed parallel to the axis of the knee joint.

Anterior trunk flexion was assessed with the child in orthostatic position, with the knees passively extended, followed by forward flexing of the trunk. Hypermobility was defined as present when the child could touch the floor with the palms of his/her hands (Table 1).

**Table 1:** Criteria, parameters and Beighton et al.^3^ scores for defining joint hypermobility

Joint	variable	definition of hypermobility	score
1. Metacarpophalangeal joints of the fifth fìnger	passive dorsiflexion of the fifth metacarpophalangeal joint	angle > 90°	2
2. Wrists	apposition to the flexor aspect of the ipsilateral forearm	touch flexed thumb to forearm	2
3. Elbows	hyperextension of the elbow	angle > 10°	2
4. Knees	hyperextension of the knee	angle > 10°	2
5. Spine	active anterior flexion of the trunk with passive extension of knees	place palms of hands flat on the floor	1

Apart from the anterior flexion of the trunk, all of the variables were analyzed bilaterally. The children performed the tests in underwear and barefoot.

The chi-squared test was used for statistical analysis to assess independence in crossreference tables, to compare results obtained from boys and girls in different age groups and to evaluate the relationship between the right and left sides. Student's t test was used to evaluate the differences between the right and left sides, by subtracting the angular values found on the left side from the ones found on the right side, and also to compare the two sides using the two-tail method. Variance analysis (ANOVA) was used to compare mean values of angle, age and gender, and to assess homogeneity. The significance level adopted was 5%.

## RESULTS

The absolute and percentage frequencies for boys and girls with scores of 0, 1 and 2 for each of the variables analyzed are shown in Table 2. Wrist flexion was the variable with highest mobility, as shown by hyperflexion in apposition of the thumb to the forearm (76.2%), followed by hyperextension of the fifth finger (53.3%) and hyperextension of the elbow (33.6%). The trunk and the knee were the joints with lowest hypermobility. Only 14% had hyperflexion of the trunk and 12.5% had hyperextension of the knee. The frequencies of the results for the variables of apposition of thumb to forearm, extension of knee and flexion of trunk differed between girls and boys, but this was not observed for the variables of extension of finger and extension of elbow. Cases of unilateral hyperextension were observed for each of the variables, but there was only a statistically significant difference in knee assessments (0.02 < p < 0.05). Nevertheless, hyperextension was found to be predominantly bilateral.

**Table 2:** Scores for lack of hypermobility (0), unilateral hypermobility (1), or bilateral hypermobility (2) in absolute and percentage frequencies for boys, girls and the total sample, for each of the variables, and chi-squared test for comparison between genders

Variable	score	boys		girls		Total	
		n	%	n	%	n	%
EXTENSION OF FIFTH FINGER	0	196	36.7	182	31.0	378	33.8
	1	68	12.7	77	13.2	145	12.9
	2	270	50.6	327	55.8	587	53.3
		χ^2^ = 4.11	df = 2		0.10 < p < 0.20		
APPOSITION OF THUMB TO FOREARM	0	123	23.0	84	14.3	207	18.5
	1	27	5.1	32	5.5	59	5.3
	2	384	71.9	47	80.2	854	76.2
		χ^2^ = 14.05	df = 2		p < 0.001		
EXTENSION OF ELBOW	0	305	57.1	325	55.5	630	56.2
	1	49	9.2	65	11.0	114	10.2
	2	180	33.7	196	33.5	376	36.6
		χ^2^ = 1.15	df = 2		0.50 < p < 0.70		
EXTENSION OF KNEE	0	461	86.3	445	76.0	906	81.0
	1	24	4.5	50	8.5	74	6.5
	2	49	9.2	91	15.5	140	12.5
		χ^2^ = 19.65	df = 2		p < 0.001		
FLEXION OF TRUNK	0	489	91.6	474	81.0	963	86.0
	1	45	8.4	112	12.0	157	14.0
		χ^2^ = 26.47	df= 1		p < 0.001		

In the analysis of the distributions of absolute and percentage frequencies of overall individual scores (0 to 9) for boys and girls, the chi-squared test was used to evaluate the independence between score and gender. A significant association was observed (χ^2^ = 35.39; degrees of freedom, df = 9; p < 0.001). There were higher rates of boys with lower scores (0 to 2) and girls with higher scores (7 to 9).

When joint hypermobility was evaluated in children according to the criteria suggested by Beighton et al.,^3^ 64.6% of the sample (60% of boys and 68.8 % of girls) had scores of 4 or greater and were, thus, considered hypermobile.

The incidence of hypermobility according to age and gender groups usually decreases with increasing age. For fifth finger extension, the association was only significant among the boys (0.02 < p < 0.05). For knee extension, there was significance both among the girls (0.02 < p < 0.05) and in the total sample (0.001 < p < 0.01). There was a highly significant association for the apposition of the thumb, regardless of gender, and also for the distribution of scores for hyperflexion of the trunk, both among the girls and in the total sample (p < 0.001). There was a statistically significant decrease in hypermobility with increasing age, even within the age range of 4 to 7 years (χ^2^ = 19.66; df = 3; p < 0.001).

## DISCUSSION

Joint mobility in human beings is complex, with few studies of normality patterns, except in relation to hypermobility, as seen from the large number of publications in the literature.

Gender and age groups are clearly associated with joint mobility. However, the criteria reported by Beighton et al.,^3^ which have been broadly accepted, were used in mixed samples, regardless of age groups and gender. This may have influenced their results, especially those relating to children of less than seven years old.

In the present study, similar numbers of boys and girls were included, and the conclusion reached was that, even at kindergarten age, girls have greater joint mobility. We have not found references in literature that can explain the reason for this difference, but the body composition of girls, with higher body fat and water, may favor mobility, whereas boys have more muscles, which may account for their lower joint mobility.

Some studies have concluded that joint mobility decreases as age increases, due to increased diameters of muscle fibers, as well as because of the decrease in water content and increase in cross-linking among the molecules.^2,19^ Most investigators have included children, teenagers and adults in their samples, and almost all of them have concluded that joint mobility decreases as age increases.^3,13^ Beighton et al.,^3^ Wordsworth et al.,^6^ Binns^29^ and Grahame^33,34^ observed that joint mobility decreases faster during childhood and more slowly among adults.

Studies evaluating age groups similar to those in the present study, i.e. preschool children, have been carried out by Carter and Wilkinson,^24^ who identified hypermobility in 7% of a sample of children aged 5 to 14 years, and Beighton et al.,^3^ who found this in 6% of boys and 20% of girls, in a sample of Africans aged zero to 19 years. Silverman et al.^35^ identified hypermobility in 10.5% of children aged 5 to 10 years, while Subramanyam and Janaki^12^ found hypermobility in 65% of boys and 35% of girls, in a sample of children aged 6 to 10 years. The present study, with children aged 4 to 7 years, found a hypermobility rate of 64.6%. In general, similar studies that included children of the same age as in the present study have had different results, probably due to the small numbers of children included.

The review by Murray and Woo^20^ shows a joint hypermobility prevalence of 5% to 30% among children, with the inclusion of other age ranges as well. In our study, 64.6% of the children were hypermobile, which may be explained by the narrow age range. Our results suggest that this narrow age range requires other methodological parameters and criteria to characterize joint mobility. The objective of the present study was to obtain more conclusive data, by only including children with slight variations in age.

Wordsworth et al.^6^ evaluated hypermobility in 248 normal individuals with ages ranging from 8 to 70, and observed that 11.5% had scores of greater than or equal to 4. Harinstein et al.^36^ found a Gaussian distribution of laxity among 500 Chinese females with ages ranging from 2 to 90. They did not characterize the different age groups, and children may have had little influence on the characterization of the sample.

These studies did not either provide full information on their objectives, such as the quantification of each gender stratum, the type of equipment used, the analysis by age group and other data that might have had a major influence on the results. Other studies did not report the numbers of individuals per age group.^32,35^ For instance, Rikken-Bultman et al.^30^ included 252 individuals aged 4 to 13 years in one of the study groups and found a hypermobility rate of 15.5%. However, the ratio of children to teenagers is not known.

Considering that differences between the left and right sides have been found for some parameters^32,35,37^ and that, in the present study, the variable of knee extension showed clear asymmetry, we suggest that bilateral evaluation should always be carried out.

Only a few studies have evaluated school children^20,35^ and none of them exclusively evaluated kindergarten children, thus making it difficult to compare results.

A total score of 4 or higher was found in 64.6% of children in the present study. The question this poses is whether most of them will be hypermobile as adults. If hypermobility decreases as age increases,^32^ the most likely answer is no.

The analysis of each parameter shows interesting results. For example, for the extension of the fifth finger, the category of a 90° angle was most frequent. An ability to perform apposition of the thumb to the forearm was also a very common finding among children. Therefore, these two variables seem inadequate for diagnosing generalized joint hypermobility among children.

On the other hand, elbow and knee extension and trunk flexion, which were observed in 33.6%, 12.5% and 14% of the individuals, respectively, could be used for diagnosing generalized joint hypermobility among preschool children. The angles found for elbow and knee extension were similar to those described in the literature.

Grahame^34^ proposed that hypermobility is a generalized collagen disorder. In the present study, we observed that most of the children had generalized joint hypermobility, which suggests that, for the majority of children, this is probably a transient period rather than a genetic condition.

The prevalence of high total scores in boys and girls, suggests that if the child has immature joint contours, greater mobility is favored. Moreover, mobility is also enhanced by the fiber capsules of most of the joints evaluated in the present study, which are responsible for the greater elasticity, as well as by the mechanical characteristics of the muscles and ligaments or by the connective tissue.^13^

Hypermobility is a common finding among kindergarten children. However, from the sample analyzed in the present study, we have concluded it is probably a normal and transient condition in most of these children. It was observed that, even within this narrow age range, there was a significant difference in mobility decrease between different ages. These results corroborate those of previous studies with regard to age range^3,7^ and the decrease in mobility as age increases, and with regard to gender, with greater mobility observed among females.^3-5^ On the other hand, the literature studied is contradictory when stating that hypermobility is a common finding among children,^11,12^ if we consider that these studies used the same methodological criteria and parameters for identifying hypermobility, for all age groups. This may also be taking place in daily clinical practice. The authors believe that other criteria should be used, in addition to the current scoring system, to evaluate joint mobility as an abnormal finding among children. The criteria should be adjusted according to age groups.

## CONCLUSIONS

None of the parameters can be used separately in preliminary screening tests for this age group, since the finding of hypermobility in one joint does not imply that it is present in other joints.

The variables of elbow and knee extension and anterior flexion of the trunk seem adequate for identifying hypermobility in this age group. However the variables of apposition of the thumb to the forearm and extension of the fifth finger seem inadequate, because they are a common finding in this age group.

We observed that joint hypermobility is a common condition in this age group and that a definitive assessment cannot be obtained on the basis of the scoring systems currently used. On the other hand, none of the parameters should be used alone in initial screenings.^38^ Therefore, new methodological criteria and parameters should be developed for identifying hypermobility among preschool children.
